# Analysis of microbial diversity of traditional Chinese starters and screening of special flavor yeast for Chinese baked flatbread

**DOI:** 10.3389/fnut.2025.1755466

**Published:** 2026-01-14

**Authors:** Yixi Yang, Yuanhui Wang, Boyu Li, Chen Chen, Yixian Zhao, Lulu Pan, Restituto Tocmo, Guoqiang Zhang, Qidong Zhang, Huang Zhang, Juntao Sun

**Affiliations:** 1 College of Food Science and Engineering, Henan University of Technology, Zhengzhou, China; 2 Department of Food and Nutritional Sciences, University of Reading, Whiteknights, Reading, Berkshire, United Kingdom; 3 Henan Province Wheat-flour Staple Food Engineering Technology Research Centre, Henan University of Technology, Zhengzhou, China; 4 School of International Education, Henan University of Technology, Zhengzhou, China; 5 College of Food Engineering, Xinxiang Institute of Engineering, Xinxiang, Henan, China; 6 School of Science, Coventry University, Coventry, United Kingdom; 7 Zhengzhou Tobacco Research Institute of CNTC, Zhengzhou, China; 8 College of Food and Biological Engineering, Henan University of Animal Husbandry and Economy, Zhengzhou, China; 9 Food and Pharmacy College, Xuchang University, Xuchang, China

**Keywords:** Chinese baked flatbread, high throughput sequencing technology, microbial community structure, traditional Chinese starters, volatiles

## Abstract

**Introduction:**

Traditional Chinese starters have unclear microbial complexity and community functions.

**Methods:**

To screen functional yeasts for flavor enhancement of Chinese baked flatbread (CBF), Saccharomyces cerevisiae Y4 was isolated and identified from traditional Chinese starters using a combined approach of high-throughput sequencing and culturomics. The traditional starters samples were aseptically collected from Luohe, Xinxiang in Henan province and Rizhao in Shandong province.

**Results:**

The analysis of microbial communities revealed that Luohe starter had the highest fungal (Chao1 = 271.86) and bacterial (Chao1 = 341.76) richness, with dominant genera including Saccharomyces and Lactiplantibacillus. Saccharomyces cerevisiae Y4 showed the following performance: saturated gas production at 48 h, 12% ethanol tolerance, high acid tolerance (OD value below 0.15), and 1975 mL gas production in fermented dough. CBF fermented by Y4 had a high sensory score (82.8) and 40 volatiles, including the unique 1-octen-3-ol and a prominent level of phenethyl acetate content.

**Discussion:**

In this study, a superior yeast strain was identified, providing theoretical insights and valuable microbial resources for optimizing the flavor of CBF.

## 1 Introduction

Chinese baked flatbread (CBF), a regionally distinctive fermented staple food, is primarily manufactured from wheat flour through dough preparation, proofing, and baking processes. The annual production of CBF in China reached 2.64 billion pieces in 2024 indicating its substantial market presence. Since the flavor quality of CBF directly influences consumers’ purchase intention and repurchase behavior, in-depth research on its flavor profile holds significant practical significance and application value. The type of starter culture employed significantly influences the final flavor and quality attributes of CBF (1). Although traditional starters require a longer fermentation time (6–72 h), staple foods fermented with traditional Chinese starters exhibit superior aroma and taste compared to those produced using pure yeast fermentation, potentially attributed to the microbial diversity inherent in conventional cultures, and microbial communities produce richer flavor substances through synergistic effects (2).

Despite advancements in microbial isolation techniques, the microbial diversity in traditional starters and the mechanisms underlying the formation of volatile flavor substances remain unclear (3). With the deepening of research, the inherent limitations of single technical systems have been overcome by more studies, which combine traditional microbial community analysis methods with modern sequencing technologies to elucidate the microbial diversity inherent in starter microbiota (4). The combination of the two enables cross-validation of data (*κ* > 0.85) and cross-regional sampling, enhancing the classification accuracy, credibility, and reproducibility of statistical data on microbial diversity and revealing region-specific microbiomes (5).

High-throughput sequencing (HTS), which has the characteristic of high speed, throughput, and accuracy, provides a powerful tool for deciphering microbial diversity in traditional sourdough fermentation (3). Gas chromatography–mass spectrometry (GC–MS) has the characteristic of high sensitivity, high resolution and strong anti-interference ability, which has been used for separating, identifying, and quantifying volatile and semi-volatile compounds in complex mixtures and offers a favorable means for exploring the volatile components of traditional starters (6). Their combination can complement for the limitations of each method individually and enable targeted screening of functional strains in traditional sourdoughs.

In this study, based on these considerations, an integrated approach combining HTS and culturomics was employed to investigate the microorganisms in three traditional Chinese starters previously screened, which were collected from Luohe, Xinxiang, and Rizhao. The microbial community structures and diversity in these starters were revealed. Simultaneously, the impact on the volatile substances of CBF was explored via solid-phase microextraction-gas chromatography–mass spectrometry (SPME-GC–MS).

## 2 Materials and methods

### 2.1 Materials

Three traditional Chinese starters were collected from Yuanhui District, Luohe City (LH), Hongqi District, Xinxiang City (XX) in Henan Province, and Donggang District, Rizhao City (RZ) in Shandong Province. Wheat flour was purchased from Henan Jinyuan Grain and Oil Co., Ltd., Zhengzhou, China, and conformed to the national standard GB/T 1355-2021 for first-grade wheat flour. Highly active dry yeast (AQ) was purchased from Angel Yeast Co., Ltd., Yichang, China.

Glucose, yeast extract powder, agar powder, tryptone, magnesium sulfate, manganese sulfate, peptone, beef extract, calcium carbonate, triammonium citrate, sodium acetate, yeast extract, Tween 80, tris, hydrochloric acid, isoamyl alcohol, isopropanol, glacial acetic acid, bromocresol green, ferric chloride, calcium chloride, potassium dihydrogen phosphate, 1,2-dichlorobenzene, potassium chloride, and anhydrous ethanol were supplied by Tianjin Kemiou Chemical Reagent Co., Ltd. The 50 × TAE Buffer, agarose, DNA Marker, 2 × Power Taq PCR Master Mix, and Taq PCR Master Mix were obtained from Sangon Biotech (Shanghai) Co., Ltd. The C_7_ - C_30_ *n*-alkane mixture standard was acquired from Sigma-Aldrich (Shanghai) Trading Co., Ltd.

### 2.2 Determination of microbial diversity in traditional starters

Fungal and bacterial DNA were extracted from the starters using the E. Z. N. A™ Mag-Bind Soil DNA Kit (Omega Bio-tek, Norcross, GA, United States, Cat. No. D3370-01), and the concentration and purity of the extracted DNA were analyzed. For fungal DNA, the internal transcribed spacer (ITS) region was amplified with the extracted fungal DNA as the template using primers SSU 0817F (5’-TTAGCATGGAATAATRRAATAGGA-3′) and SSU 1196R (5’-TCTGGACCTGGTGAGTTTCC-3′). For bacterial DNA, the V3-V4 hypervariable region was amplified with the extracted bacterial DNA as the amplification template using primers 338F (5’-ACTCCTACGGGAGGCAGCAG-3′) and 806R9 (5’-GGACTACHVGGGTWTCTAAT-3′). The purified amplicons were quantified, and then sequenced on the Illumina MiSeq PE300 platform. The sequencing results were uploaded to the National Center for Biotechnology Information (NCBI) database. Usearch was used to conduct operational taxonomic unit (OTU) clustering analysis on sequences with > 97% similarity (excluding singletons), and chimeras were removed to obtain representative OTU sequences. The optimal alignment results for the sequences were selected using an alignment threshold of 90%.

### 2.3 Isolation, purification, and preservation of advantageous yeast and bacteria

#### 2.3.1 Isolation and purification of yeast and lactic acid bacteria

Appropriately diluted solutions (200 μL) were spread on WL medium (for yeast) and MRS medium [for lactic acid bacteria (LAB)], which were then incubated at 30 °C and 36 °C for 2–3 days, respectively. Single colonies were randomly picked based on differences in colony morphology on the media and re-streaked for isolation. After incubation, pure single colonies of yeast and LAB were obtained.

#### 2.3.2 Strain preservation

Single colonies were picked for activation and culture. Then, glycerol and bacterial suspension were mixed at a 1:1 ratio in cryovials, which were stored in a − 80 °C refrigerator for later use.

### 2.4 Yeast and bacterial DNA extraction

Yeast genomic DNA was extracted using the E. Z. N. A.® Yeast DNA Kit (Omega Bio-tek, D3370-01) following the manufacturer’s protocol: enzymatic pre-lysis with lyticase (30 min, 37 °C), bead-beating with 0.5 mm glass beads (FastPrep-24, 6.0 m·s^−1^, 30 s × 3), followed by silica-column binding and elution in 50 μL elution buffer.

Bacterial DNA was obtained using the DNeasy UltraClean Microbial DNA Kit (Qiagen, 12,224–50) according to the manufacturer’s instructions: samples were resuspended in MicroBead solution, mechanically lysed in a bead-beater, treated with MD1/MD2 lysis buffers (65 °C, 10 min), and finally purified on silica spin filters with MD4 wash buffer and eluted in 100 μL MD5 buffer.

### 2.5 Gene amplification

Using the extracted yeast and bacterial DNA as templates, the yeast ITS region was amplified with primers ITS4 (5’-TCCTCCGCTGACTAATATGC-3′) and ITS5 (5’-GGAAGTAAAAGTCGTAACAAGG-3′), and the bacterial 16S rRNA gene was amplified with primers P0 (5’-GAGAGTTTGATCCTGGCTCAG-3′) and P6 (5’-CTACGGCTACCTT-3′).

PCR was conducted using a PTC Genesy 96 T thermal cycler (Xi’an Tianlong Technology Co., Ltd.). All amplifications were performed in 25.5 μL reactions with four replicates. Each reaction volume contained 1 μL of DNA template, 1 μL of Primer ITS4/P0, 1 μL of Primer ITS5/P6, 12.5 μL of 2 × Power Taq PCR Master Mix (Thermo Scientific, United States) and 10 μL of Double-distilled water.

The following cycle parameters were used for yeast: initial denaturation for 5 min at 94 °C; 30 cycles of 30 s at 94 °C (denaturation), 45 s at 52 °C (annealing), and 45 s at 72 °C (extension); and final extension for 10 min at 72 °C.

The following cycle parameters were used for bacteria: initial denaturation for 5 min at 94 °C; 30 cycles of 60 s at 94 °C (denaturation), 60 s at 52 °C (annealing), and 90 s at 72 °C (extension); and final extension for 10 min at 72 °C.

### 2.6 Electrophoresis detection

Electrophoresis analysis was performed using a 0.8% agarose gel with a DNA Marker as reference, under conditions of 100 V voltage, 100 mA current, and 25 min duration using an electrophoresis apparatus (Model YJ300C, Beijing Junyi Dongfang Electrophoresis Equipment Co., Ltd., Beijing, China). After staining with ethidium bromide solution for 40 min, the PCR amplification fragments were observed under an ultraviolet analyzer (UVP LLC, Upland, CA, United States) to determine their clarity, and the results were photographed and recorded.

### 2.7 Gene sequencing

The PCR products were sent to Sangon Biotech (Shanghai) Co., Ltd. for sequence determination. Homology search was then performed in the NCBI BLAST database to identify the species of the tested strains.

### 2.8 Activation and collection of microbial strains

Yeast and LAB were streaked onto YPD medium and MRS medium, separately, and incubated at 28 °C and 36 °C for 48 h. Single colonies were then picked up and inoculated into YPD liquid medium and MRS liquid medium, respectively. After 20 h of cultivation, the supernatant was discarded to obtain fresh wet cells of yeast and LAB.

### 2.9 Yeast fermentation capacity

The fermentation capacity of yeast was determined using the Durham tube fermentation method. Activated yeast was inoculated into YPD liquid medium containing an inverted Durham tube, followed by incubation at 28 °C for 48 h. The volume of gas in the Durham tube was observed every hour, and the gas production initiation time of the strain and the gas production status at 48 h were recorded.

### 2.10 Lactate, acetate, and ethanol tolerance of yeast

Yeast seed culture (100 μL) was inoculated into YPD liquid media with different acid and ethanol concentrations, followed by shaking cultivation at 28 °C and 180 r/min for 20 h. The absorbance was then measured at a wavelength of 600 nm. The absorbance value was used to represent the growth density of yeast under different acid and ethanol concentrations, thus reflecting the acid tolerance and ethanol tolerance of the yeast. The volume ratios (v/v) of lactic acid and acetic acid added were as follows: (0 + 0) %, (0.4 + 0.1) %, (0.8 + 0.2) %, (1.2 + 0.3) %, (1.6 + 0.4) %, and (2 + 0.5) %. The addition amounts of ethanol were 0, 5, 10, and 12%, respectively.

### 2.11 Gas production characteristics of yeast-fermented dough

Fresh wet yeast cells were inoculated into flour at a concentration of 10^8^ CFU/g for dough mixing, and the gas production properties of the dough were determined using a Chopin F3 rheological fermentation instrument (Chopin Technologies, Villeneuve-la-Garenne, France). The test conditions were set as follows: temperature 35 °C; determination duration 3 h; weight of the counterbalance 1.5 kg.

### 2.12 Preparation of yeast-fermented CBF

Fresh wet yeast cells were added to 200 g of wheat flour at an inoculum size of 10^7^ CFU/g, with 48% water addition for dough mixing. After forming a dough with a smooth and elastic surface, it was placed in a proofing box for the first fermentation (temperature: 30 °C; relative humidity: 85%; duration: 6 h). After proofing, wheat flour (100 g) and water (45 mL) were added for remixing into dough, which was then repeatedly rolled out to fully expel air. The dough was subsequently divided into 100 g portions, which were shaped into round dough blanks using a round dough press, and then placed in the proofing box for the second fermentation (temperature: 30 °C; relative humidity: 85%; duration: 40 min). The proofed dough blanks were then transferred to an oven for baking with the top heat set to 180 °C and the bottom heat to 200 °C, for 16 min. After baking, the products were naturally cooled at room temperature for 30 min, and subsequently stored in a sealed container for later use.

### 2.13 Sensory evaluation of CBFs

The sensory evaluation was carried out by 20 trained panelists (10 males and 10 females, 20–40 years old) in an isolated sensory booth (room temperature 20–22 °C). The panelists were trained according to ISO standards (ISO 8586: 2012) for sensory analysis and subjected to performance monitoring (ISO 11132: 2012). Compusense software (version 5.2, Compusense Business Avionics B. V., Canada) was used for sample distribution. Panelists finished the sensory evaluation within 10 min and rinsed their mouths with spring water between samples. The appearance, color, palatability, toughness, viscosity, smoothness, and flavor of the CBF was scored by panelists on the basis of Supplementary Table S1.

### 2.14 Determination of volatiles

Volatiles were extracted from CBF samples using headspace solid-phase microextraction (HS-SPME) and analyzed by Gas chromatography–mass spectrometry (GC–MS) instrument (Model 8,890-5977B, Agilent Technologies (China) Co., Ltd., China) with a DB-5 capillary column (60 m × 0.25 mm × 0.25 μm) (7).

1. Solid-phase microextraction conditions: CBF sample (2 g) was placed into a 20 mL headspace vial and internal standard solution (1 μL, 1,2-dichlorobenzene, 100 mg/L) was immediately added. Sampling was performed using an automatic headspace injection. The extraction head was aged for 30 min, the incubator was preheated at 60 °C for 10 min, the extraction time was 40 min, and the sample was desorbed at 250 °C for 300 s at the injection port.
2. GC–MS conditions: The temperature program was as follows: initial temperature 40 °C held for 3 min, then 3 °C/min increase to 60 °C, followed by 4 °C/min increase to 200 °C, and finally 5 °C/min increase to 260 °C held for 10 min; injection volume: 1 μL; flow rate 1.0 mL/min; no split; full scan mode.
3. Qualitative analysis: The mass spectra obtained using GC–MS data analysis software were compared with the NSIT. L 17 spectral library. The retention index (RI) values of the detected compounds were calculated using the retention times of *n*-alkanes (C_7_–C_30_) and compared with the database (https://webbook.nist.gov/).
4. Quantitative analysis: Semi-quantification of volatiles was performed using the internal standard method. Following the procedure:
$$C_{X}=\frac{S_{X}}{S(\text{internal})}\times \frac{C(\text{internal})\times V(\text{internal})\;}{M(\text{sample})\;}$$

In Formula, *C_X_* is the relative mass concentration of compound X in the CBF sample, μg/kg; *S_X_* is the peak area of compound X in the CBF sample; *S(internal)* is the peak area of the internal standard 1,2-dichlorobenzene; *C(internal)* is the concentration of the internal standard 1,2-dichlorobenzene; *V(internal)* is the volume of the internal standard added; *M(sample)* is the mass of the sample, g.

### 2.15 Statistical analysis

All experiments were performed in triplicate, and the results were expressed as “mean ± standard deviation.” For significant difference analysis, one-way analysis of variance (ANOVA) and Duncan’s multiple range test (*p* < 0.05) were used, with statistical analysis conducted using IBM SPSS Statistics 25 (IBM Corporation, Armonk, NY, United States). Graphs were plotted using Origin Pro 2025 (OriginLab Corporation, Northampton, MA, United States).

## 3 Results and discussion

### 3.1 Alpha diversity analysis of traditional Chinese starters microbial communities

Alpha diversity analysis, a crucial method for evaluating microbial community complexity, reflects the species richness and evenness of samples (8). As shown in Table 1, for both fungi and bacteria in the traditional starter samples of RZ, XX, and LH, the Coverage indices were all greater than 99%, indicating that the sequencing results were reliable. The LH starter sample exhibited significantly higher species richness in both fungi (Chao1 = 271.86; ACE = 274.36) and bacteria (Chao1 = 341.76; ACE = 351.64) than the other samples. This value range is consistent with the species richness characteristics of traditional dough starters reported in existing studies which detected 490 fungal OTUs and 234 bacterial OTUs in Chinese northwest traditional jiaozi (9). The high species richness of LH may be linked to the combination of its unique raw material composition or fermentation processes and potential cross-kingdom synergistic effects (8).

**Table 1:** Alpha diversity of different traditional Chinese starters.

Microorganisms	Sample	Chao1 index	Shannon index	Simpson index	Ace index	Sobs index	Coverage index /%
Fungi	RZ	195.27 ± 11.33^b^	1.75 ± 0.33^b^	0.38 ± 0.11^a^	194.63 ± 9.87^c^	158 ± 5.03^b^	99.94 ± 0.02^ab^
	LH	271.86 ± 17.26^ab^	1.94 ± 0.54^ab^	0.35 ± 0.06^a^	274.36 ± 13.45^ab^	271 ± 7.95^ab^	99.95 ± 0.01^b^
	XX	203.11 ± 13.27^b^	2.29 ± 0.61^ab^	0.22 ± 0.05^ab^	208.89 ± 8.22^c^	178 ± 5.66^b^	99.96 ± 0.01^b^
Bacteria	LH	341.76 ± 20.17^a^	2.84 ± 0.85^a^	0.11 ± 0.01^c^	351.64 ± 10.97^a^	337 ± 9.17^a^	99.83 ± 0.05^a^
	XX	332.66 ± 14.25^a^	1.23 ± 0.17^b^	0.50 ± 0.13^a^	339.23 ± 14.11^a^	325 ± 4.51^a^	99.82 ± 0.06^a^
	RZ	301.86 ± 10.92^a^	2.25 ± 0.39^a^	0.22 ± 0.07^b^	306.13 ± 12.85^a^	300 ± 3.69^a^	99.83 ± 0.02^a^

LH, RZ and XX represent the traditional Chinese starters from Luohe, Rizhao and Xinxiang. Different uppercase letters in the same column indicate significant differences at *p <* 0.05 level.

In contrast, although the fungal richness in the XX starter was moderate, its Shannon index (2.29) and Simpson index (0.22) indicated that the fungal community possessed higher evenness and niche differentiation capacity. This aligns with the findings of which reported that traditional sourdoughs with balanced microbial evenness often establish stable metabolic interaction networks (10). Such high evenness might arise from a more complex metabolic interaction network in its fermentation environment, which is also supported by the findings that high niche differentiation in traditional fermented foods contributes to functional stability (8, 11). Giving that the Coverage indices of all samples exceeding 99%, the Sobs index (271) of the LH fungal community was almost consistent with the Chao1 estimate (271.86). This consistency not only confirms the accuracy of the sequencing data but also suggests that rare microbial groups (abundance < 0.1%) in LH were fully captured. Rare taxa, despite their low abundance, often harbor unique functional genes that may play crucial roles (9). This effective detection provides a valuable dataset to explore uncharacterized microbial functions in traditional starters.

### 3.2 Fungal and bacterial community structures in traditional Chinese starters

**Figure 1:**
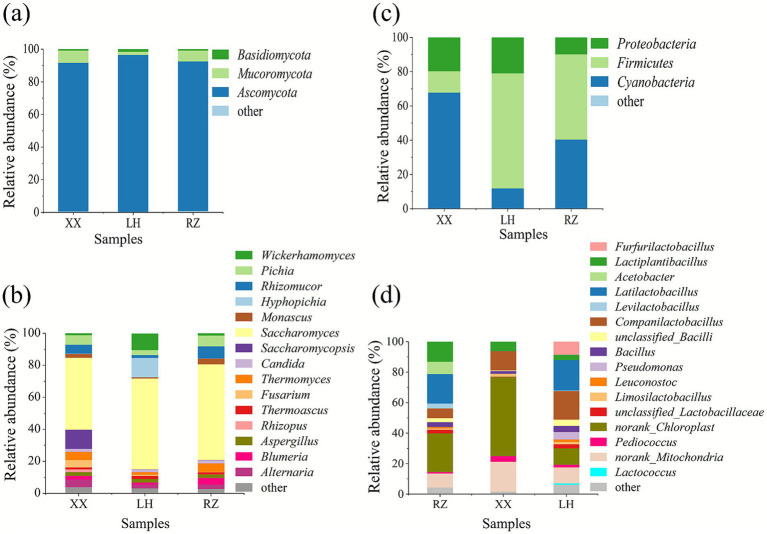
Fungal and bacterial community structures in traditional Chinese starters. **(a)** Species composition at the phylum level of fungi in the traditional Chinese starters. **(b)** Species composition at the genus level of fungi in the traditional Chinese starters. **(c)** Species composition at the phylum level of bacteria in the traditional Chinese starters. **(d)** Species composition at the genus level of bacteria in the traditional Chinese starters. LH, RZ, and XX represent the traditional Chinese starters from Luohe, Rizhao, and Xinxiang.

Analysis of the relative abundance of fungal community composition is recognized as a critical method for uncovering the proportional distribution of distinct fungal species within a community (12). The relative abundance of fungal communities in XX, LH, and RZ starters are shown in Figures 1a,b. At the phylum level, three identifiable fungal phyla were detected: *Ascomycota*, *Mucoromycota*, and *Basidiomycota*. Among these, *Ascomycota* had the highest relative abundance (RA: > 1%), in XX (RA: 91.11%), LH (RA: 96.18%), and RZ (RA: 91.71%) starters, respectively. At the genus level, 15 fungal genera with relative abundances greater than 1% were detected.

*Saccharomyces*, as the dominant genus within *Ascomycota*, exhibited extremely high relative abundances in samples XX (46.98%), LH (56.83%), and RZ (59.86%). *Saccharomyces* is the primary microbial group involved in dough fermentation, exerting significant influence on dough expansion height and gluten network structure (13). Furthermore, *Saccharomycopsis* has an excellent ability to produce *β*-glucosidase, acid protease, and amylase, which helps promote microbial community succession enriching the flavor of malt (14). Previous studies have shown that bread fermented with exhibits superior flavor properties and lower hardness, as the genus *Wickerhamomyces anomalus* can convert precursor substances into aromatic substances such as esters, acids, alcohols, and aldehydes via synthetases (15).

Moreover, *Saccharomyces* exhibited the highest relative abundance in RZ sample, indicating that RZ starter possessed superior fermentation capacity. In addition, the main dominant fungal genera in XX sample also include *Saccharomycopsis*, *Pichia*, *Rhizomucor*, and *Thermomyces*. In LH sample, the main dominant fungal genera were *Hyphopichia* (RA: 12.12%) and *Wickerhamomyces*, (RA: 10.30%). In RZ sample, the main dominant fungal genera were *Rhizomucor* and *Pichia*.

Notably, *Aspergillus*, *Monascus Rhizopus*, and *Rhizomucor* were detected in XX, LH, and RZ samples. These are important fermentation strains in mold culture, possessing strong saccharification enzyme activity, which can provide the fermentation agent with multiple enzyme systems, supplying a large amount of small-molecule monosaccharides and free amino acids in the fermentation agent for metabolic utilization by lactic acid bacteria and yeast (16).

Analysis of the relative abundance of bacterial community composition is recognized as a critical method for uncovering the proportional distribution of distinct bacterial species within a community, and the structural characteristics of dominant and rare taxa can be reflected (12). The relative abundances of bacterial communities in XX, LH, and RZ starters are shown in Figures 1c,d. At the phylum level, three bacterial phyla were detected: *Firmicutes*, *Cyanobacteria*, and *Proteobacteria*, and they are commonly found in traditional Chinese starters (17). At the genus level, 12 dominant bacterial genera were detected. The dominant bacterial genera in XX sample were *Companilactobacillus* (RA: 12.55%) and *Lactiplantibacillus* (RA: 6.00%). The main dominant bacterial genera in RZ sample were *Latilactobacillus* (RA: 19.35%)*, Lactiplantibacillus* (RA: 12.97%), and *Acetobacter* (RA: 8.10%). In LH sample, the main dominant bacterial genera were *Companilactobacillus* (RA: 18.71%), *Latilactobacillus* (RA: 19.98%), and *Lactiplantibacillus* (RA: 8.47%). *Companilactobacillus*, *Latilactobacillus*, and *Lactiplantibacillus* produce lactic acid and other organic acids during dough fermentation, which enrich the flavor of fermented bread products (18). *Acetobacter* was the only dominant bacterial genus in RZ sample, and it oxidizes ethanol to produce acetic acid during dough fermentation and improves the texture and taste of bread products (14).

### 3.3 Isolation, purification, and molecular identification of yeast and bacterial strains

Based on morphological differences such as colony shape, color, size, and protrusion degree, a total of 94 yeast strains were selected from XX, LH, and RZ starters, with their colony morphologies shown in Supplementary Table S2, exhibiting seven distinct colony morphologies. Through isolation, purification, analysis, and identification, species composition within microbial communities can be recognized, relative abundances and distribution characteristics of different bacteria in the community determined, and interactions among microorganisms explored (19).

The ITS sequence is extensively applied in species identification and widely recognized as the “barcode” region for fungal classification (20). The gel electrophoresis detection results of partial yeast strains are presented in Supplementary Figure S1a. Target fragments were successfully amplified from all samples. The fragment size was consistent with the expected ITS region length (500–1,000 bp), indicating that the primer design and amplification conditions exhibited favorable specificity and effectiveness.

The molecular identification results of the yeast strains are shown in Table 2. Three yeast species were identified from the three starters (XX, LH, and RZ): *Saccharomyces cerevisiae*, *Wickerhamomyces anomalus*, and *Hyphopichia burtonni*. Strains Y1, Y2, Y3, Y4, and Y5 were all identified as *Saccharomyces cerevisiae* through molecular identification, and their colony morphology characteristics differed. This is because the colony morphology exhibited by *Saccharomyces cerevisiae* is related to intraspecific genetic diversity (21).

**Table 2:** Molecular identification of yeast and bacteria in traditional Chinese starters.

Name	Strains identified	Colony morphology	Number of times identified		
			RZ	XX	LH
Y1	*Saccharomyces cerevisiae*	I	10.3 ± 1.2^a^	13.5 ± 1.3^a^	9.5 ± 0.9^a^
Y2	*Saccharomyces cerevisiae*	II	4.5 ± 0.7^b^	4.1 ± 0.2^c^	8.3 ± 0.6^a^
Y3	*Saccharomyces cerevisiae*	III	7.8 ± 0.7^ab^	4.0 ± 0.1^c^	3.2 ± 0.1^b^
Y4	*Saccharomyces cerevisiae*	IV	3.1 ± 0.8^b^	6.8 ± 1.4^ab^	3.1 ± 0.2^b^
Y5	*Saccharomyces cerevisiae*	V	4 ± 0.6^b^	6 ± 0.7^b^	0
Y6	*Wickerhamomyces anomalus*	VI	0	0	4.7 ± 0.3^ab^
Y7	*Hyphopichia burtonii*	VII	0	2.2 ± 0.1^bc^	4.1 ± 0.5^ab^
L1	*Furfurilactobacillus rossiae*	/	3.7 ± 0.2^b^	0	0
L2	*Latilactobacillus curvatus*	/	0	0	4.7 ± 0.1^ab^
L5	*Lactiplantibacillus plantarum*	/	2.1 ± 0.5^c^	6.5 ± 0.3^ab^	7.7 ± 1.1^a^
L7	*Acetobacter malorum*	/	3.0 ± 0.6^b^	0	3.6 ± 0.1^b^
L8	*Companilactobacillus crustorum*	/	5.2 ± 1.0^ab^	6.1 ± 0.8^b^	3.7 ± 0.2^b^

I, II, III, IV, V, VI, VII, VII are correspond, respectively, to description of yeast in traditional Chinese starters. LH, RZ and XX represent the traditional Chinese starters from Luohe, Rizhao and Xinxiang. Different uppercase letters in the same column indicate significant differences at *p <* 0.05 level.

During dough fermentation, *Saccharomyces cerevisiae* primarily metabolizes various sugars to produce CO₂, causing the dough to rise, and can also undergo esterification reactions through metabolic processes, enriching the flavor of fermented foods (13). Moreover, *Saccharomyces cerevisiae* can impart good flavor to steamed breads and produce substances that inhibit fungal growth during dough fermentation and bread storage (15). *Wickerhamomyces anomalus* possesses excellent capabilities for producing various glycosidases, including *β*-D-glucosidase and *α*-L-arabinofuranosidase, and is widely present in traditional sourdough and mold culture (22).

The results of gel electrophoresis detection for partial bacterial strains are presented in Supplementary Figure S1b. Fragment sizes were mainly concentrated in the range of 1,000–2000 bp. The sizes of the fragments were consistent with the typical length of bacterial 16S rRNA genes, verifying the reliability of the amplification products.

The molecular identification results for the three species of bacteria from XX, LH and RZ are shown in Table 2. A total of four LABs and one acetic acid bacterium (*Acetobacter malorum*) were identified. The four LABs were *Furfurilactobacillus rossiae*, *Latilactobacillus curvatus*, *Lactiplantibacillus plantarum*, and *Companilactobacillus crustorum*. *Lactiplantibacillus plantarum* is a common LAB in traditional Chinese starters, capable of producing large amounts of organic acids during dough fermentation, which positively improves the quality of fermented dough products (18). *Acetobacter malorum* is an obligate aerobic bacterium that metabolizes ethanol produced by yeast fermentation to generate acetic acid and water, positively influencing the flavor of fermented dough products made with traditional Chinese starters (23).

### 3.4 Functional characterization of yeast

#### 3.4.1 Yeast fermentation capacity analysis

Glucose, maltose, sucrose, and other carbohydrates in wheat flour serve as the sole carbohydrate sources for yeast, among the most critical characteristics of high-quality yeast is dough fermentation capacity (24). The fermentation capacity of yeast strains Y1–Y7 was determined using the Durham tube fermentation method. As shown in Table 3, within 48 h of fermentation, the Durham tubes of all five *Saccharomyces cerevisiae* (Y1–Y5) were filled with gas, indicating that these five strains possess strong fermentation capabilities. This performance aligns with the key characteristics of *Saccharomyces cerevisiae* reported in traditional fermented food studies (23). In contrast, some studies isolated non-*Saccharomyces* yeasts from traditional Chinese dough starters, which showed rapid initial gas production (within 3 h) but lower total gas yield, highlighting the superior long-term fermentation stability of our *Saccharomyces cerevisiae* strains (25). Among them, *Saccharomyces cerevisiae* Y4 began gas production at 6 h of fermentation, with the earliest fermentation initiation time, indicating the best fermentation initiation ability.

**Table 3:** The fermentation ability of yeast and volumetric and sensory scores of CBFs made from different yeasts.

Strain	Fermentation time (h)	Gas production at 48 h	Specific volume (mL/g)	Sensory score
Y1	7	+++	1.99 ± 0.01^b^	81.10 ± 1.0^b^
Y2	7	+++	1.98 ± 0.02^b^	81.00 ± 1.0^b^
Y3	6	+++	2.01 ± 0.02^ab^	82.40 ± 0.5^ab^
Y4	6	+++	2.02 ± 0.01^ab^	82.80 ± 0.5^a^
Y5	8	+++	1.98 ± 0.02^b^	81.70 ± 0.6^ab^
Y6	22	++	1.94 ± 0.01^c^	81.00 ± 0.8^b^
Y7	24	++	1.92 ± 0.02^c^	79.20 ± 0.7^c^
AQ	0.5	+++	2.01 ± 0.01^ab^	81.90 ± 0.8^ab^

“++” indicates that the gas production of the strain is approximately 3/4 of the volume of the Durham tubes; “+++” indicates that the Durham tubes is filled with gas. The samples for specific volume (mL/g) and sensory score refer to the CBF prepared using the corresponding strains. Y1–Y5 represent Saccharomyces cerevisiae Y1–Y5. Y6 represents Wickerhamomyces anomalus Y6. Y7 represents Hyphopichia burtonni Y7. Different uppercase letters in the same column indicate significant differences at *p* < 0.05 level.

#### 3.4.2 Lactic acid and acetic acid tolerance of yeast

**Figure 2:**
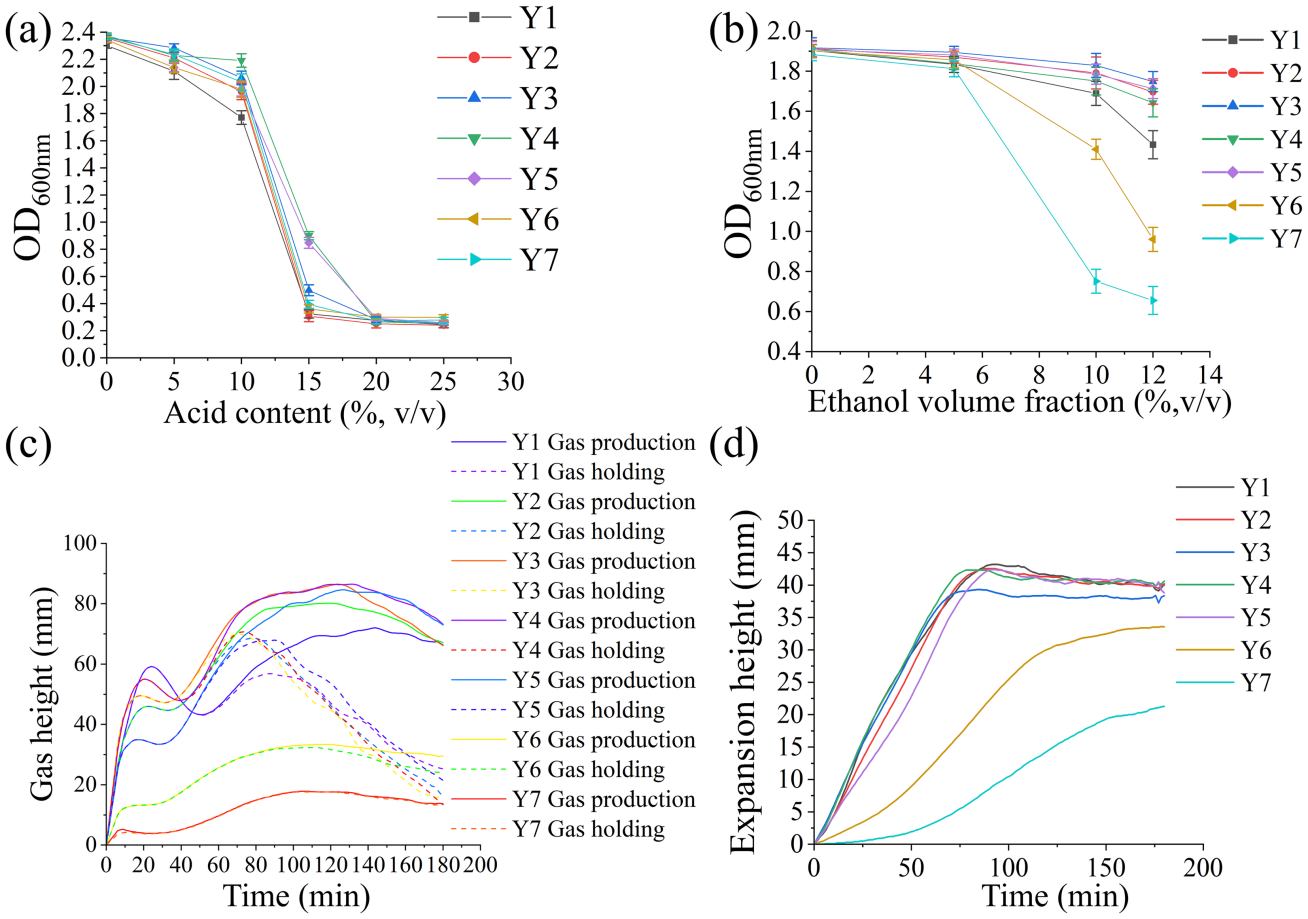
**(a)** Acid tolerance of yeast. **(b)** Ethanol tolerance of yeast. **(c)** Gas release curves of doughs fermented with different yeast strains. **(d)** Ferment height curves of doughs fermented with different yeast strains. Y1–Y5 represent *Saccharomyces cerevisiae* Y1–Y5. Y6 represents *Wickerhamomyces anomalus* Y6. Y7 represents *Hyphopichia burtonni* Y7.

During dough fermentation, the fermentation characteristics of yeast are influenced by extracellular environmental factors such as high temperature, low pH, and high ethanol concentration (21). The lactic acid and acetic acid tolerance of yeast strains Y1–Y7 were determined, with yeast growth density expressed by optical density (OD). As shown in Figure 2a, with the increase of acid content, the OD values of the seven yeast strains gradually decreased, reaching a stable state at an acid content of 20% (v/v). At an acid content of 15% (v/v), the OD values of *Saccharomyces cerevisiae* Y4 and Y5 were significantly higher than those of *Saccharomyces cerevisiae* Y1, Y2, Y3, *Wickerhamomyces anomalus* Y6, and *Hyphopichia burtonni* Y7, indicating that *Saccharomyces cerevisiae* Y4 and Y5 had better tolerance to lactic acid and acetic acid. Moreover, differences exist in acid tolerance among *Saccharomyces cerevisiae*, which is consistent with previous research (23). Furthermore, the pH during dough fermentation typically ranges from 3.8 to 6.7. Therefore, all seven yeast strains can adapt to the acidic environment of dough, and *Saccharomyces cerevisiae* Y4 and Y5 exhibit superior acid tolerance.

#### 3.4.3 Ethanol tolerance of yeast

During dough fermentation, yeast produces ethanol through carbohydrate metabolism, and excessively high ethanol concentrations can reduce yeast activity and inhibit its proliferation rate (21). The ethanol tolerance of *Saccharomyces cerevisiae* Y1, Y2, Y3, Y4, Y5, *Wickerhamomyces anomalus* Y6, and *Hyphopichia burtonni* Y7 were determined (Figure 2b). When the ethanol volume fraction exceeded 10%, the OD values of all seven yeast strains began to decrease, indicating that the growth was inhibited. *Saccharomyces cerevisiae* Y1–Y5 exhibited stronger ethanol tolerance than *Wickerhamomyces anomalus* Y6 and *Hyphopichia burtonni* Y7. All seven yeast strains could tolerate an ethanol volume fraction of 10% (v/v), and the ethanol concentration in fermented dough generally did not affect their activity and proliferation rate.

#### 3.4.4 Gas production characteristics of yeast fermentation dough

Yeast aerobic fermentation generates abundant gas, which enables the full stretching of the gluten network and retains CO₂ in the dough to form gas pockets, thereby endowing fermented dough with a soft texture (26). As shown in Figure 2c, significant differences were observed in gas productions and gas retention capacities of doughs fermented by seven yeast strains (Y1–Y7). For doughs fermented by *Saccharomyces cerevisiae* Y1–Y5, gas production first increased sharply within the initial 40 min of fermentation, then decreased significantly, followed by a slow increase to the maximum gas production. Furthermore, the gas production of the fermented dough of *Saccharomyces cerevisiae* Y4 was the highest (total gas production of 1975 mL).

As shown in Figure 2d, the expansion heights of the doughs fermented by *Saccharomyces cerevisiae* Y1–Y5 first increased continuously and then stabilized or decreased slightly. The expansion heights of the doughs fermented by *Saccharomyces cerevisiae* Y1, Y2, Y4, Y5 showed no significant differences, with maximum expansion heights of 43.2 mm, 41.9 mm, 42.3 mm, and 42.4 mm, respectively, and they were significantly greater than that of *Saccharomyces cerevisiae* Y3 (35.2 mm). Furthermore, the expansion heights of the doughs fermented with *Wickerhamomyces anomalus* Y6 and *Hyphopichia burtonni* Y7 continued to increase slowly. The results showed that *Saccharomyces cerevisiae* Y4 can quickly initiate the gas production process and significantly shorten the dough fermentation time. Moreover, it steadily maintained a high height with minimal fluctuations, which can ensure the stability of the dough structure and reduce the risk of collapse caused by a sudden drop in gas production. Based on the above results, it can be concluded that the dough fermented with *Saccharomyces cerevisiae* Y4 has strong gas production and retention capabilities, resulting in better dough fermentation quality.

### 3.5 Sensory evaluation analysis of yeast fermentation for making CBF

Sensory evaluations of CBFs made with seven yeast strains (Y1–Y7) were conducted, and AQ yeast was as the control. As shown in Table 3 and Supplementary Table S3, CBF fermented individually by the seven yeast strains showed no significant differences in surface color and surface morphology that the sensory scores of these CBFs ranged 79.20–82.80. CBFs fermented with the five *Saccharomyces cerevisiae* exhibited larger specific volume, better springiness, fine and uniform internal pores, soft texture, crisp taste, and no stickiness to the teeth. *Saccharomyces cerevisiae* Y4 exhibited remarkable advantages, and the CBF fermented by this strain showed higher volumetric ratios, indicating stronger gas-producing and dough expansion capacities. Moreover, it achieved the highest overall sensory score (82.80 ± 0.5) and performed well in multiple dimensions. In contrast, Y6 and Y7 had lower sensory scores (81.00 ± 0.8 and 79.20 ± 0.7). The sensory score (81.90 ± 0.8) of control AQ performed on par with most *Saccharomyces cerevisiae* strains lower than Y4.

### 3.6 Volatiles of CBFs made by *Saccharomyces cerevisiae* Y4 and yeast (AQ)

The composition of volatiles in baked food serves as the fundamental material basis for reflecting the aroma characteristics of baked food (7). As shown in Table 4, a total of 40 volatiles were detected in the CBF (Y4) sample, including alcohols (12), esters (5), aldehydes (10), acids (6), ketones (2), furan (1), and other compounds (4). CBF (Y4) and CBF (AQ) samples had 38 and 36 volatiles, respectively.

**Table 4:** Compositions of volatiles in CBFs made by *Saccharomyces cerevisiae* Y4 and Angel yeast (AQ).

Compound	RI^a^	RI^b^	Relative content of volatiles (μg/kg)		Identification^c^
			Y4	AQ	
Isobutanol	654	654	0.85 ± 0.12	8.5 ± 0.75	MS, RI
Isopentanol	744	743	22.01 ± 2.34	30.71 ± 2.4	MS, RI
2-Methylbutanol	741	740	19.67 ± 1.12	20.18 ± 3.1	MS, RI
2,3-Butanediol	804	806	6.2 ± 0.23	3.9 ± 0.16	MS, RI
*n*-Hexanol	874	872	4.7 ± 0.33	5.48 ± 0.33	MS, RI
*n*-Heptanol	970	970	2.01 ± 0.13	1.79 ± 0.21	MS, RI
1-Octen-3-ol	980	981	1.68 ± 0.11	—	MS, RI
2-Ethylhexanol	1,026	1,025	0.63 ± 0.09	0.74 ± 0.11	MS, RI
Octanol	1,078	1,078	1.69 ± 0.16	2.09 ± 0.17	MS, RI
Phenethyl alcohol	1,118	1,117	40.58 ± 3.21	39.87 ± 3.09	MS, RI
cis-3-Non-1-enol	1,157	1,156	7.56 ± 0.34	13.72 ± 0.32	MS, RI
trans-5-Decenol	1,247	1,247	3.55 ± 0.12	—	MS, RI
Alcohols			111.13 ± 15.21	126.98 ± 16.02	MS, RI
*n*-Hexanal	819	819	2.81 ± 0.18	3.06 ± 0.1	MS, RI
Heptanal	901	901	1.56 ± 0.15	1.79 ± 0.13	MS, RI
(*E*)-2-Heptenal	957	960	0.94 ± 0.05	0.69 ± 0.1	MS, RI
Benzaldehyde	966	965	15.32 ± 1.23	15.44 ± 1.47	MS, RI
*n*-Octanal	1,002	1,003	0.66 ± 0.07	0.5 ± 0.08	MS, RI
Phenylacetaldehyde	1,044	1,044	6.67 ± 0.37	6.69 ± 0.71	MS, RI
trans-2-Octenal	1,055	1,056	1.86 ± 0.1	1.88 ± 0.17	MS, RI
Nonanal	1,104	1,102	11.42 ± 0.58	12.66 ± 0.7	MS, RI
trans-2-Nonanal	1,163	1,163	5.69 ± 0.2	4.73 ± 0.41	MS, RI
Decanal	1,205	1,205	2.88 ± 0.19	3.87 ± 0.27	MS, RI
Aldehydes			49.81 ± 3.89	51.71 ± 4.12	MS, RI
Ethyl octanoate	1,192	1,193	5.55 ± 0.62	9.4 ± 0.26	MS, RI
Ethyl phenylacetate	1,245	1,243	10.51 ± 1.09	4.27 ± 0.3	MS, RI
Ethyl nonanoate	1,320	1,319	—	1.42 ± 0.1	MS, RI
Propyl nonanoate	1,365	1,365	6.43 ± 0.31	5.72 ± 0.12	MS, RI
Ethyl decanoate	1,389	1,392	4.73 ± 0.22	8.73 ± 0.19	MS, RI
Esters			27.22 ± 1.11	29.54 ± 0.91	MS, RI
Isovaleric acid	847	848	0.54 ± 0.13	0.24 ± 0.05	MS, RI
2-Methylbutanoic acid	855	854	0.88 ± 0.12	0.61 ± 0.1	MS, RI
Hexanoic acid	990	990	3.11 ± 0.18	2.85 ± 0.2	MS, RI
Benzoic acid	1,170	1,170	3.28 ± 0.15	2.94 ± 0.2	MS, RI
Octanoic acid	1,180	1,182	3.41 ± 0.11	8.63 ± 0.24	MS, RI
N-nonanoic acid	855	854	9.93 ± 0.31	8.09 ± 0.34	MS, RI
Acids			21.15 ± 1.33	23.36 ± 0.91	MS, RI
3-Hydroxy-2-butanone	744	743	1.78 ± 0.13	4.41 ± 0.17	MS, RI
Geranyl acetone	1,448	1,449	3.56 ± 0.2	4.81 ± 0.26	MS, RI
Ketones			5.34 ± 0.31	9.22 ± 0.41	MS, RI
2-*n*-pentylfuran	990	990	5.43 ± 0.17	5.51 ± 0.3	MS, RI
Furans			5.43 ± 0.11	5.51 ± 0.25	MS, RI
Naphthalene	1,180	1,179	1.16 ± 0.15	—	MS, RI
Valencene	1,493	1,495	1.65 ± 0.12	2.2 ± 0.21	MS, RI
Indole	1,292	1,292	4.66 ± 0.31	—	MS, RI
Others			7.47 ± 0.35	3.7 ± 0.19	MS, RI

‘—’ indicates not detected in the sample; the relative content units (μg/kg) of volatiles are calculated using a semi-quantitative method. Y4 represents *Saccharomyces cerevisiae* Y4. AQ represents Angel yeast.

a RIs were calculated against n-alkanes C_7_–C_30_ on DB-5 column.

b RIs (DB-5 column) were compared with the database (https://webbook.nist.gov/).

c In qualitative methods, MS stands for mass spectrometry comparison, and RI stands for gas phase retention index.

During dough fermentation, yeast metabolizes amino acids via the Ehrlich pathway to generate various alcoholic metabolites, which exert a positive impact on the herbal and wine-like aromas of fermented products (27). CBF (Y4) contained high relative contents of phenethyl alcohol, 2-methylbutanol, and isopentanol, consistent with previous research findings. Phenethyl alcohol has a rose-like aroma and is positively correlated with bread aroma, while 2-methylbutanol can add rich, fruity, and floral aromas to fermented dough products (28). Isoamyl alcohol, derived from leucine metabolism, is positively correlated with aroma and exhibits “fragrant, mellow, and malt-like” flavors (29). Compared to CBF (AQ), CBF (Y4) uniquely contained 1-octen-3-ol and trans-5-decenoic alcohol, and the former contributed mushroom and grassy aromas to add a fresh layer of complexity, while the latter further enriched the stereochemical structure of alcohol compounds (7). Moreover, relative content of isobutyl alcohol in CBF (Y4) was significantly lower than in CBF (AQ), avoiding a harsh alcoholic taste (28).

Furthermore, aldehydes are produced via the oxidation of lipids and amino acids. Among the detected aldehyde volatiles, benzaldehyde, nonanal, phenylacetaldehyde, *n*-hexanal, and trans-2-nonenal are the main ones, characterized by flavor profiles of nutty, floral, and fruity aromas (7). Meanwhile, acidic compounds not only produce acidity during dough fermentation but also serve as important precursors for other aromatic compounds, positively contributing to flavor formation (30). The acidic volatiles detected in CBF (Y4) primarily included caproic acid, benzoic acid, and caprylic acid. An appropriate amount of caproic acid can contribute a cheesy flavor with an oily aroma (27). Caprylic acid exhibits a mild cheesy taste and can react with ethanol to form caprylic acid ethyl ester, imparting a fruity aroma to CBF (Y4).

Esters, as important aromatic compounds, are formed via the esterase-catalyzed reaction between unsaturated fatty acids and alcohols during fermentation (31). Among ester volatiles, those with relatively high contents include ethyl caprylate, phenethyl acetate, *γ*-nonalactone, and ethyl decanoate. The content of ethyl phenylacetate in CBF (Y4) is 2.46 times that of CBF (AQ), contributing a fruity aroma to CBF (29). In addition, both 3-hydroxy-2-butanone and geranyl propionate were detected in two CBF samples. Ketones are mainly produced through amino acid degradation and *β*-oxidation of saturated fatty acids (18). 3-Hydroxy-2-butanone contributes a buttery aroma, while geranyl propionate provides a fruity aroma. 2-Pentyl furan imparts a floral aroma to CBF (Y4). The unique indole in CBF (Y4) further enhance the uniqueness of the flavor. The results showed that CBF (Y4) had abundant unique volatiles, synergistically enhancing flavor complexity and uniqueness.

## 4 Conclusion

Seven yeast strains were isolated and obtained from three traditional Chinese starters, and *Saccharomyces cerevisiae* Y1–Y5 significantly outperformed *Wickerhamomyces anomalus* Y6 and *Hyphopichia burtonni* Y7 in terms of fermentation capacity, alcohol tolerance, and gas production. Moreover, the fermentation characteristics values of *Saccharomyces cerevisiae* Y4 are the best among the five *Saccharomyces cerevisiae* strains. CBF made with Y4 exhibited more balanced metabolic characteristics in terms of the types and concentrations of key volatiles (1-octen-3-ol, trans-5-decen-1-ol, 2-phenylethanol, phenethyl acetate, and trans-2-nonenal), and it ensured a rich diversity of volatiles and avoided excessive production of irritating components. In the future, the growth and metabolic dynamics of microorganisms and the complex interactions between them during the dough fermentation process will be further investigated.

## Data Availability

The original contributions presented in the study are included in the article/Supplementary material, further inquiries can be directed to the corresponding authors. The original contributions presented in the study of the 16S rRNA gene sequences are publicly available. This data can be found here: https://www.ncbi.nlm.nih.gov/search/, nucleotide sequence(s): PX775921-PX775937,PX775938-PX775947.
