# Supplementary material for: Analysis of microbial diversity of traditional Chinese starters and screening of special flavor yeast for Chinese baked flatbread
Source: Front Nutr. 2026 Jan 14;12:1755466. doi: 10.3389/fnut.2025.1755466 (PMC12849762; doi:10.3389/fnut.2025.1755466)
Supplement: Supplementary file 1 [file Table_1.DOCX]

Supplementary Material

# Supplementary Figures and Tables

## Supplementary Figures


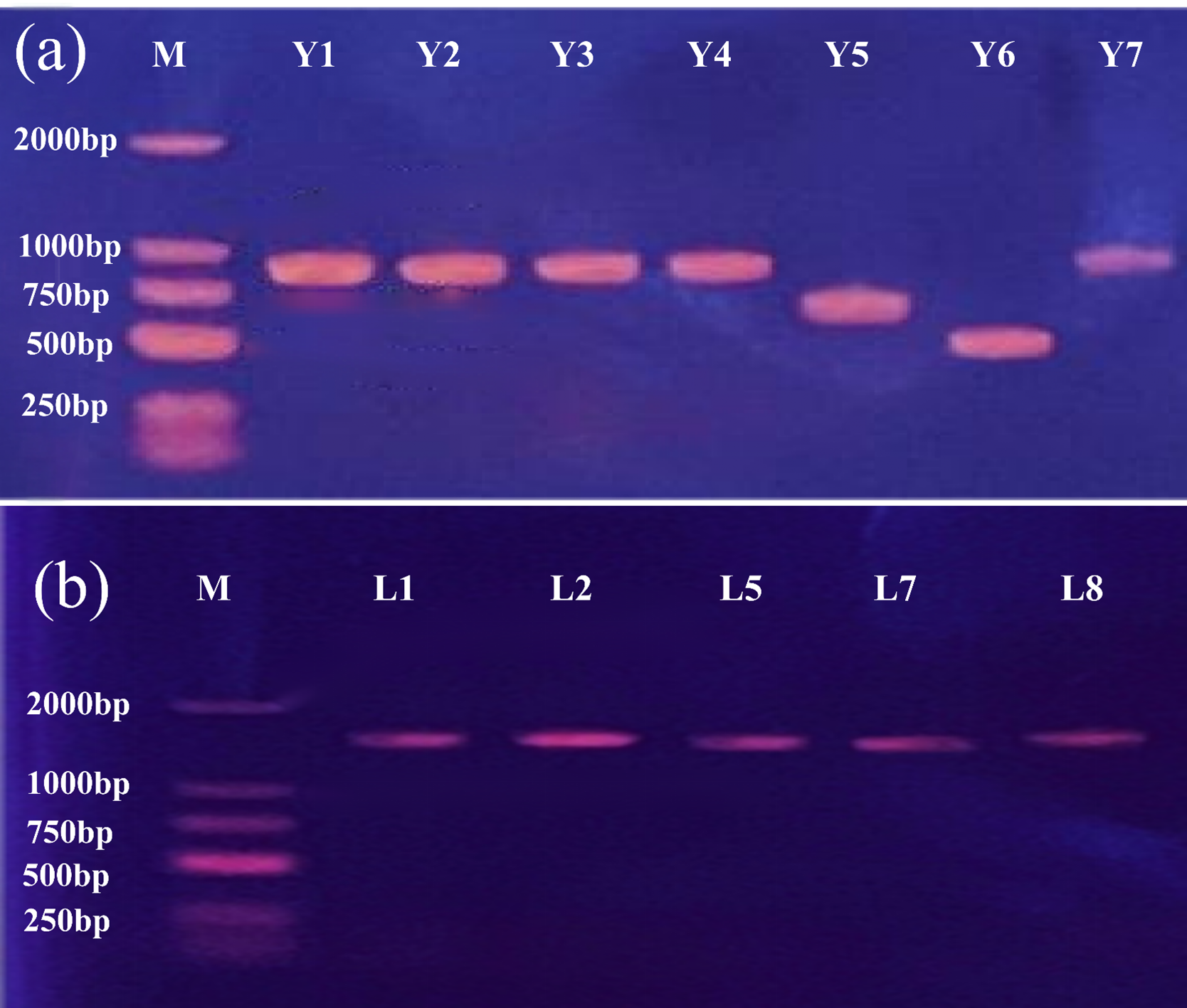


**Supplementary Figure 1.** (a) Electrophoresis of PCR amplified products of some bacteria 16S sequences. (b) Electrophoresis of PCR amplified products of some yeast ITS sequences. M represents DNA Marker. Y1 ~ Y5 represent *Saccharomyces cerevisiae* Y1 ~ Y5. Y6 represents *Wickerhamomyces anomalus* Y6. Y7 represents *Hyphopichia burtonni* Y7. L1 represents *Furfurilactobacillus rossiae*. L2 represents *Latilactobacillus curvatus.* L5 represents *Lactiplantibacillus plantarum*. L7 represents *Companilactobacillus crustorum*. L8 represents *Acetobacter malorum*.

## Supplementary Tables

**Supplementary Table S1. Sensory evaluation of CBF**

| Project | Total Score | Scoring Criteria |
| --- | --- | --- |
| Specific Volume | 15 | Specific volume > 2.1 mL/g: deduct 1 point for every 0.1 mL/g increase;  Specific volume 1.8–2.1 mL/g: 15 points;  Specific volume < 1.8 mL/g: minimum score of 5 points |
| Surface Color | 10 | Uniform color, golden yellow or slightly yellow: 8–10 points;  Basically uniform color, whitish, dark yellow, or with small dark spots: 5–7 points;  Uneven color, excessive dark spots: 0–4 points |
| Surface Shape | 15 | Complete shape, uniform thickness, no cracks: 12–15 points;  Fairly intact shape with slight wrinkles, bubbles, or cracks: 8–11 points;  Collapsed, rough, and asymmetrical: 0–7 points |
| Internal structure | 15 | Small, uniform pores, fine texture, no crumbling: 12–15 points;  Pores are relatively uniform, structure is slightly coarse, and no obvious crumbling is present: 8–11 points;  Pores are uneven, structures are very coarse, and dryness, hardness, and crumbling are present: 0–7 points |
| Viscosity and elasticity | 15 | Good rebound elasticity, crisp and not sticky to the teeth: 12–15 points;  Weak rebound or slightly sticky to the teeth: 8–11 points;  Dry, hard, very weak rebound, or very sticky to the teeth: 0–7 points |
| Flavor | 15 | Sweet and aromatic: 11–15 points;  Mild flavor: 6–10 points;  Burnt taste or other off-flavors: 0–5 points |
| Odor | 15 | Fermented aroma with no off-odors: 11–15 points;  Weak or no fermented aroma: 6–10 points;  Rancid or other off-odors: 0–5 points |

**Supplementary Table S2 Description of yeast in** **traditional Chinese starters**

| Classification | Colony morphology | RZ | XX | LH | Colony morphology appearance |
| --- | --- | --- | --- | --- | --- |
| Ⅰ | Green protruding part, white edge | ＋ | ＋ | ＋ | 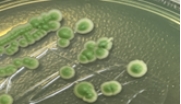 |
| Ⅱ | White protruding part, green edge | ＋ | ＋ | ＋ | 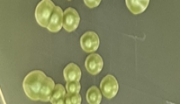 |
| Ⅲ | Round, dark green | ＋ | ＋ | ＋ | 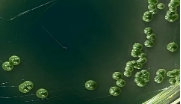 |
| Ⅳ | Milky white, grey-green pointed protruding part | ＋ | ＋ | ＋ | 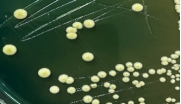 |
| Ⅴ | Raised portion is scattered green | ＋ | ＋ | - | 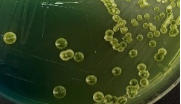 |
| Ⅵ | Edges are light blue, round | - | - | ＋ | 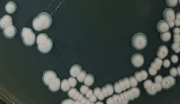 |
| Ⅶ | White, with wrinkles on the surface | - | ＋ | ＋ | 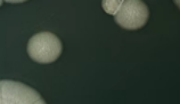 |

Note: “+” indicates that this starter contains colonies of this morphology; “-” indicates that this starter does not contain colonies of this morphology. LH, RZ and XX represent the traditional Chinese starters from Luohe, Rizhao and Xinxiang.

**Supplementary Table S3 Volumetric and sensory scores of CBF made from different yeast**

| Project | Specific Volume | Surface Color | Surface Shape | Internal structure | Viscosity and elasticity | Flavor | Odor | Total Score |
| --- | --- | --- | --- | --- | --- | --- | --- | --- |
| Y1 | 15.00 | 8.10±0.8^c^ | 12.40±0.9^c^ | 12.30±0.7^b^ | 12.10±0.8^b^ | 10.60±0.3^a^ | 10.60±0.9^c^ | 81.10±1.0^b^ |
| Y2 | 15.00 | 8.20±0.3^a^ | 12.30±0.2^a^ | 12.20±0.5^ab^ | 12.10±0.7^b^ | 10.60±0.5^ab^ | 10.60±0.8^c^ | 81.00±1.0^b^ |
| Y3 | 15.00 | 8.30±0.1^a^ | 12.40±0.1^a^ | 12.40±0.2^a^ | 12.10±0.3^a^ | 11.00±0.1^a^ | 11.20±0.2^a^ | 82.40±0.5^ab^ |
| Y4 | 15.00 | 8.20±0.5^b^ | 12.30±0.5^b^ | 12.40±0.2^a^ | 12.30±0.3^a^ | 11.30±0.2^a^ | 11.30±0.2^a^ | 82.80±0.5^a^ |
| Y5 | 15.00 | 8.10±0.6^b^ | 12.30±0.5^b^ | 12.30±0.1^a^ | 12.20±0.2^a^ | 10.80±0.3^a^ | 11.00±0.1^a^ | 81.70±0.6^ab^ |
| Y6 | 15.00 | 8.20±0.3^a^ | 12.30±0.6^b^ | 12.10±0.4^ab^ | 11.80±0.7^b^ | 10.60±0.4^ab^ | 11.00±0.3^b^ | 81.00±0.8^b^ |
| Y7 | 15.00 | 8.10±0.1^a^ | 12.30±0.5^b^ | 11.80±0.2^a^ | 11.40±0.1^a^ | 10.30±0.5^ab^ | 10.30±0.3^b^ | 79.20±0.7^c^ |
| AQ | 15.00 | 8.20±0.1^a^ | 12.30±0.4^b^ | 12.40±0.1^a^ | 12.40±0.5^b^ | 10.80±0.1^a^ | 10.80±0.1^a^ | 81.90±0.8^ab^ |

Note: The samples for sensory score refer to the CBF prepared using the corresponding strains. Y1 ~ Y5 represent *Saccharomyces cerevisiae* Y1 ~ Y5. Y6 represents *Wickerhamomyces anomalus* Y6. Y7 represents *Hyphopichia burtonni* Y7. Different uppercase letters in the same column indicate significant differences at *P* < 0.05 level.
